# MolPrompt: improving multi-modal molecular pre-training with knowledge prompts

**DOI:** 10.1093/bioinformatics/btaf466

**Published:** 2025-08-23

**Authors:** Yang Li, Chang Liu, Xin Gao, Guohua Wang

**Affiliations:** College of Computer and Control Engineering, Northeast Forestry University, Harbin 150040, China; College of Computer and Control Engineering, Northeast Forestry University, Harbin 150040, China; Computer Science Program, Computer, Electrical and Mathematical Sciences and Engineering Division, King Abdullah University of Science and Technology (KAUST), Thuwal 23955-6900, Kingdom of Saudi Arabia; Center of Excellence for Smart Health (KCSH), King Abdullah University of Science and Technology (KAUST), Thuwal 23955-6900, Kingdom of Saudi Arabia; Center of Excellence on Generative AI, King Abdullah University of Science and Technology (KAUST), Thuwal 23955-6900, Kingdom of Saudi Arabia; College of Computer and Control Engineering, Northeast Forestry University, Harbin 150040, China

## Abstract

**Motivation:**

Molecular pre-training has emerged as a foundational approach in computational drug discovery, enabling the extraction of expressive molecular representations from large-scale unlabeled datasets. However, existing methods largely focus on topological or structural features, often neglecting critical physicochemical attributes embedded in molecular systems.

**Result:**

We present MolPrompt, a knowledge-enhanced multimodal pre-training framework that integrates molecular graphs and textual descriptions via contrastive learning. MolPrompt employs a dual-encoder architecture consisting of Graphormer for graph encoding and BERT for textual encoding, and introduces knowledge prompts, semantic embeddings constructed by converting molecular descriptors into natural language, into the graph encoder to guide structure-aware representation learning. Across tasks including molecular property prediction, toxicity estimation, cross-modal retrieval, and anticancer inhibitor identification, MolPrompt consistently surpasses state-of-the-art baselines. These results highlight the value of embedding domain knowledge into structural learning to improve the depth, interpretability, and transferability of molecular representations.

**Availability and implementation:**

The source code of MolPrompt is available at: https://github.com/catly/MolPrompt.

## 1 Introduction

A comprehensive understanding of molecular properties and functions lies at the heart of rational drug discovery, forming the basis for the development of targeted therapeutics, elucidation of disease mechanisms, and the advancement of precision medicine. However, traditional discovery pipelines remain constrained by labor-intensive experimentation, prolonged development timelines, and high economic costs. These challenges have catalyzed a surge of interest in computational approaches, particularly those powered by artificial intelligence (AI), to accelerate the identification and optimization of drug candidates (Chen *et al.* 2023, Blanco-Gonzalez *et al.* 2023).

Among the most transformative developments in artificial intelligence, large-scale pre-trained models (PTMs) have substantially advanced natural language processing by learning general-purpose representations from massive unannotated corpora. Models such as BERT (Devlin 2019), GPT (Brown *et al.* 2020), and T5 (Raffel *et al.* 2020) have demonstrated exceptional generalization across a broad spectrum of downstream tasks. Motivated by their success, analogous pre-training paradigms have been increasingly adopted in molecular sciences to derive meaningful representations from molecular data, typically using SMILES strings or molecular graphs as input. These unimodal molecular PTMs have yielded encouraging results in applications such as molecular property prediction, virtual screening, and de novo molecule generation (Li *et al.* 2022, Ross *et al.* 2022, Fang *et al.* 2023, Li *et al.* 2023, Zhang *et al.* 2023).

Despite their effectiveness, unimodal approaches are fundamentally constrained by their reliance on structural information alone. In contrast, human chemists interpret molecular behavior through the integration of multiple information modalities—including structural depictions, physicochemical properties, textual descriptions from literature, and relational knowledge within biological networks. Analogously, molecules themselves are inherently multimodal entities: a single compound may be represented as a 2D or 3D graph, a natural language description, a vector of descriptors, and as a node in a biomedical knowledge graph. Capturing this multimodal richness is critical for enabling deep, interpretable, and transferable molecular understanding.

In response, recent efforts have explored multimodal molecular representation learning frameworks that unify heterogeneous information sources into cohesive molecular embeddings. For instance, KV-PLM (Zeng *et al.* 2022) enhances molecular representations by embedding SMILES strings within corresponding textual descriptions, aligning structural and linguistic features at the token level. MoMu (Su *et al.* 2022) introduces dual encoders for molecular graphs and free-text annotations, leveraging cross-modal contrastive learning to correlate semantic and structural information. Liu *et al.* (2023a) further scale this paradigm by constructing a large dataset of molecule-text pairs, enabling joint training of graph and language encoders. Expanding beyond two modalities, MolFM (Luo *et al.* 2023) integrates knowledge graphs as an additional input channel, allowing for tri-modal fusion across structural, textual, and relational knowledge spaces, thus improving model contextualization and biological relevance.

Nevertheless, current multimodal frameworks face two key limitations that hinder their ability to fully capture the semantic and functional complexity of molecular systems. First, they lack systematic integration of domain-specific knowledge, particularly chemical and physical properties, that is essential for accurately modeling molecular behavior. Second, most existing approaches treat molecular graphs and SMILES sequences as disjoint modalities, encoding them independently without explicitly modeling their intrinsic correlations. This decoupled architecture precludes early-stage interaction between topological and sequential features, thereby limiting the model’s capacity to learn fine-grained intermodal dependencies that are critical for robust and generalizable molecular representations. Taken together, these limitations underscore the urgent need for a semantically enriched, knowledge-integrated pre-training framework capable of holistically unifying structural, textual, and physicochemical modalities within a cohesive representation space.

To address these challenges, we introduce MolPrompt, a multimodal molecular pre-training framework enhanced by domain-specific knowledge prompts. Unlike prior approaches that treat molecular modalities in isolation or fuse them at later stages, MolPrompt performs early-stage interaction of structural and semantic information by transforming physicochemical descriptors into textual prompts and embedding them directly into the molecular graph encoder. This prompt-guided enrichment allows domain-specific knowledge to participate in the structural representation learning process, enhancing the semantic expressiveness of molecular embeddings. These enriched structural representations are then aligned with molecular descriptions through a cross-modal contrastive learning strategy, enabling MolPrompt to capture fine-grained intermodal correlations and generate more transferable, knowledge-informed molecular representations. Extensive evaluations across multiple benchmarks, including molecular property prediction, toxicity classification, cross-modal molecular retrieval, and anticancer target inhibitor identification, demonstrate that MolPrompt consistently outperforms state-of-the-art methods. These findings highlight the value of integrating structured domain knowledge into multimodal molecular modeling, and position MolPrompt as a promising foundation model for next-generation AI-driven drug discovery.

## 2 Materials and methods

**Figure 1.**
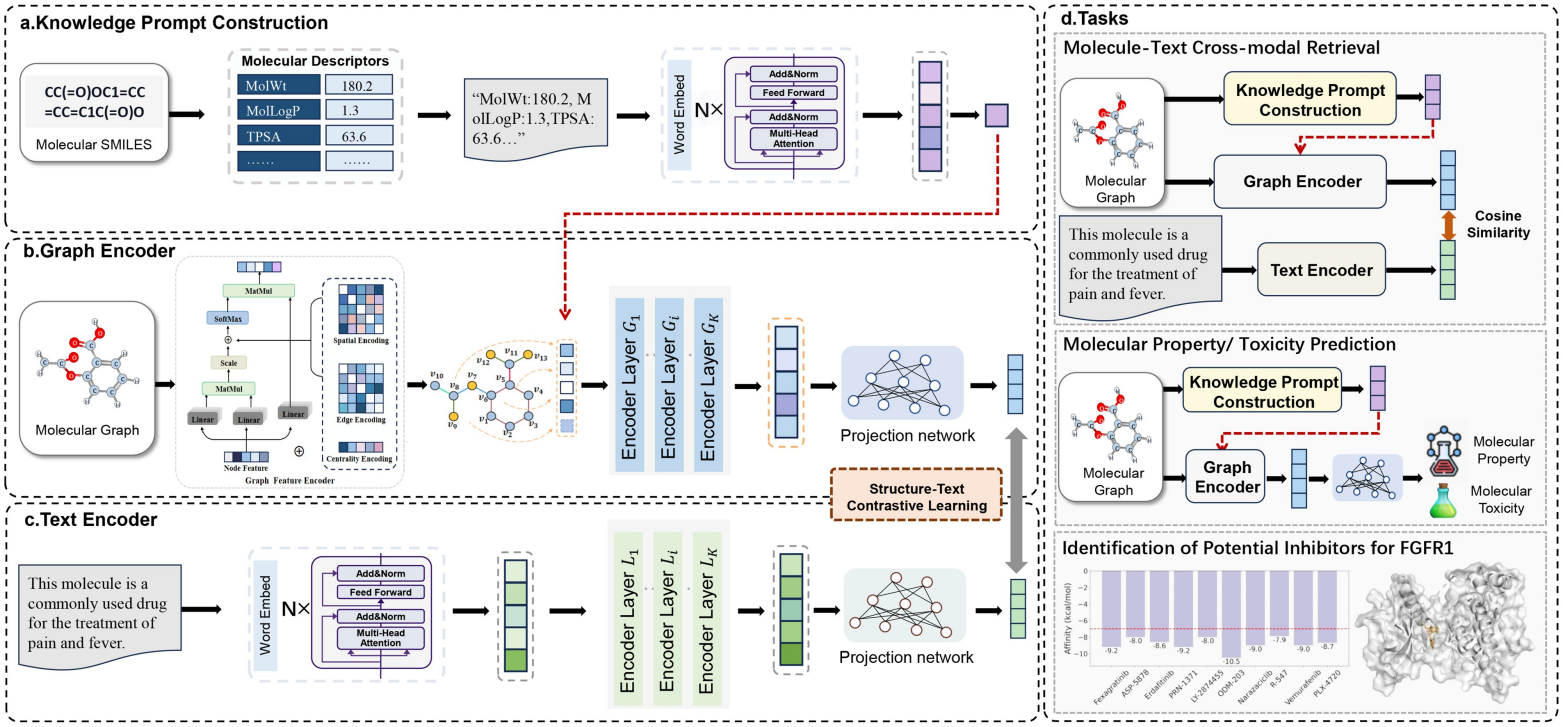
The overall architecture of MolPrompt. MolPrompt consists of four main components: knowledge prompt construction, molecular graph encoder, molecular description encoder, and cross-modal contrastive learning.

In this section, we detail the proposed multimodal molecular pre-training method with knowledge prompts, MolPrompt, which consists of four main components: knowledge prompt construction, molecular graph encoder, molecular description encoder, and cross-modal contrastive learning (Fig. 1). Initially, we extract ten commonly used molecular descriptors to create text-based molecular knowledge prompts that encapsulate the chemical and physical property information of the molecules. Next, we use distinct encoders to process molecular graph structures and molecular descriptions separately, capturing their specific representations. In particular, the knowledge prompts embed domain-specific physicochemical priors into the graph encoder, enabling it to learn structurally grounded and semantically enriched representations. By facilitating early interaction between topological features and SMILES-derived property information, MolPrompt captures intrinsic cross-modal correlations, thereby enhancing its capacity for accurate molecular representation. Finally, through a cross-modal contrastive learning framework, the two encoders are connected to jointly learn the representations of molecular graph structures and molecular descriptions.

### 2.1 Data collection

We collected approximately 200 000 molecular structure–text pairs from PubChem (Kim *et al.* 2021) to construct a large-scale molecular graph–text dataset, PC200K. Data processing details are provided in Section 1, available as supplementary data at *Bioinformatics* online.

### 2.2 Knowledge prompt construction

Knowledge can be defined as external prior information related to molecules. The existing methods for incorporating knowledge into molecular pre-training models can be divided into two categories. One category involves introducing knowledge related to elements in molecules in the form of knowledge graphs. KANO (Fang *et al.* 2023) constructs a molecular knowledge graph oriented towards chemical elements, utilizing the basic knowledge of chemical elements and their closely related functional groups. MolFM (Luo *et al.* 2023) focuses on capturing information related to molecules and other compounds, targets, and diseases through knowledge graphs. The other category is the introduction of molecular descriptors and molecular fingerprints, which are related to the physical and chemical properties of molecules (Mao *et al.* 2024), as prior knowledge. KPGT (Li *et al.* 2023) initializes molecular descriptors and molecular fingerprints as external knowledge, connecting them to the atom nodes in the molecular graph as knowledge nodes. We propose MolPrompt, which constructs molecular descriptors as text prompts. By using the same word embedding processing method as for molecular description text, we obtain knowledge embeddings and incorporate them into the structural feature embeddings of molecular graphs, thereby capturing more semantically rich representations of molecular graph structural features.

Firstly, we employ the RDkit (Bento *et al.* 2020) toolkit to extract molecular descriptors from molecular SMILES sequences. RDKit offers over 200 molecular descriptors, from which we select ten widely used descriptors as the external chemical and physical property prior knowledge of the molecules. A detailed explanation of each molecular descriptor is provided in Section 2 and Table 1, available as supplementary data at *Bioinformatics* online.

**Table 1.** Performance comparison on the PCDes and MoMu datasets for cross-modal molecule-text retrieval tasks.

	PCDes dataset		MoMu dataset	
Model	M2T R@20	T2M R@20	M2T R@20	T2M R@20
KV-PLM	75.9	64.3	0.5	0.3
MoMu-S	79.1	75.5	43.3	43.4
MoMu-K	80.2	79.0	43.7	43.5
MoleculeSTM	80.4	77.0	70.5	66.9
MolCA	85.6	82.3	76.8	73.3
**MolPrompt-w/o prompt**	**86.7**	**83.6**	**77.3**	**74.7**
**MolPrompt**	**88.2**	**86.5**	**79.4**	**76.9**

Bold values indicate our model and its variant, corresponding to the best and second-best performance, respectively.

To overcome the limitation of existing molecular graph encoders, which capture topological features but fail to incorporate semantic knowledge of molecular properties, we construct a text-based knowledge prompt by converting numerical molecular descriptors into natural language. This design integrates physicochemical priors into the graph encoder, enabling it to transcend purely topological representations and generate semantically informed molecular encodings. By facilitating early interaction between topological features and SMILES-derived property information, MolPrompt captures intrinsic cross-modal correlations that are typically overlooked in disjoint encoding schemes, thereby enhancing its capacity for accurate molecular representation. The format is unified as “Descriptor1: Value1, Descriptor2: Value2, …, Descriptor10: Value10,” where each value is rounded to one decimal place. The resulting text prompt is then fed into the BERT embedding layer to produce the knowledge prompt embedding $P_{0}=\left[p_{0}^{1},p_{0}^{2},\ldots ,p_{0}^{N_{p}}\right]\in R^{N_{p}\times d_{p}}$. As a label for the entire text semantics, the class token can represent the overall semantic meaning of the text. We extract the class (CLS) token from the knowledge prompt embedding, denoted as $h^{p}$:

(1) $$h^{p}=p_{0}^{1}.$$

### 2.3 Graph encoder

The molecular graph structure encoder of MolPrompt is designed to generate molecular representations by processing two-dimensional molecular graphs. Conventional multimodal molecular graph structure-text contrastive learning methods predominantly utilize Graph Neural Networks (GNNs) (Xu *et al.* 2018) as the molecular graph structure encoder. However, GNNs may encounter difficulties in capturing long-range interactions between atoms and lack the capability to manage complex molecular graph structures, particularly when dealing with large-scale molecular graph structures. Consequently, we employ a Graph Transformer as the molecular graph structure encoder.

Graphormer (Ying *et al.* 2021) is constructed upon the fundamental architecture of the canonical Transformer. In contrast to Graph Neural Networks, it is capable of capturing the relationships among atomic nodes within molecular graphs in a more comprehensive manner and exhibits enhanced capabilities for processing complex molecular graph structures. The Graphormer is composed of multiple Transformer layers, namely the Graphormer layer. In the Graphormer layer, the Layer Normalization (LN) is applied before Multi-Head Attention (MHA) and Feed Forward Network (FFN) instead of after. The formula is as follows:

(2) $$H^{\prime l}=\text{MHA}\left(\text{LN}\left(H^{l-1}\right)\right)+H^{l-1},$$

 

(3) $$H^{l}=\text{FFN}\left(\text{LN}\left(H^{\prime l}\right)\right)+H^{\prime l}.$$

In the above formula, $H^{l-1}$ and $H^{l}$ respectively represent the node feature matrices of the (l−1)-th and *l*-th layer in Graphormer.

Given a node feature matrix $H\;\in \;R^{n\times d_{g}}$ corresponding to a molecular graph, the computation procedure of the multi-head self-attention module in *l*-th layer of Graphormer is delineated as follows.

(4) $$Q^{l,k}=H^{l-1}W_{Q}^{l,k},\;K^{l,k}=H^{l-1}W_{K}^{l,k},\;V^{l,k}=H^{l-1}W_{V}^{l,k},$$

 

(5) $$A^{l,k}=\frac{Q^{l,k}{\left(K^{l,k}\right)}^{T}}{\sqrt{d_{K}}},\;\mathrm{Attn}\left(H^{l,k}\right)=\mathrm{softmax}\left(A^{l,k}\right)V^{l,k}.$$

where $H^{l-1}$ stands for the node feature matrix at the (l−1)-th layer, $W_{Q}^{l,k}\;\in \;R^{d_{g}\times \;d_{K}}$, $W_{K}^{l,k}\;\in \;R^{d_{g}\times \;d_{K}}$, $W_{V}^{l,k}\;\in \;R^{d_{g}\times \;d_{V}}$ stand for the trainable projection matrices of the *k*-th head at layer *l*.

Given a molecular graph G=(V,E), where $V=\{v_{1},v_{2},\ldots ,v_{n}\}$ signifies the set of nodes (i.e. atoms), and $E={\left\{e_{i,j}\right\}}_{i,j\in [1,n]}$ denotes the set of edges (i.e. chemical bonds), with *n* indicating the number of nodes. We initialize the features of nodes and edges in the molecular graph structure via RDKit. The initial features of the atom $v_{i}$ and bond $e_{i,j}$ are denoted as $x_{i}^{v}\in R^{D_{v}}$ and $x_{i,j}^{e}\in R^{D_{e}}$ respectively, where $D_{v}$ and $D_{e}$ represent the dimensions of node and edge features, respectively.

Graphormer leverages the structural information reflected on nodes and the relationships between pairs of nodes in graphs to enhance the capacity for modeling graph-structure data. Specifically, as the centrality encoding is performed on each node, it is simply added to the node features as input.

(6) $$h_{i}^{\left(0\right)}=x_{i}^{v}+z_{\deg(v_{i})},$$

where $z\in R^{d_{g}}$ are learnable embedding vectors specified by the degree $\deg(v_{i})$.

After the process represented by the above formula, we obtain the molecular graph node features $H^{0}$, $H^{0}=\left[\;h_{1}^{\left(\;0\right)}\;,h_{2}^{\left(\;0\right)}\;,\ldots ,h_{n}^{\left(\;0\right)}\right]\in R^{n\times d_{g}}$. The last node feature of $H^{0}$ is replaced with the molecular knowledge prompt embedding $h^{p}$.

(7) $$H^{0}=\left[h_{1}^{\left(0\right)},h_{2}^{\left(0\right)},\ldots ,h_{n-1}^{\left(0\right)},h^{p}\right]\in R^{n\times d_{g}}$$

The last node feature of $H^{0}$, after replacement, contains the chemical and physical prior knowledge of the molecule.

At each stage, $H^{i}$ is fed into the (i+1)-th Graphormer layer $G_{i+1}$ of the molecular graph structure encoder.

(8) $$H^{i}=G_{i}\left(H^{i-1}\right),\;i=1,2,\ldots ,K_{g}.$$

In the above formula, $K_{g}$ represents the total number of layers in the molecular graph structure encoder, $K_{g}=12$.

### 2.4 Text encoder

Molecular description text offers detailed information regarding a molecule’s composition, structure, and function. We regard molecular description text as an additional modality that corresponds to the molecular graph structure, with the objective of capturing the intricate relationships between these two modalities. Consequently, we have designed a text encoder to extract feature representations from molecular description text.

We employ the BERT model (Devlin 2019) as the text encoder, which is extensively utilized as a feature extractor in natural language processing. Molecular-related text data, especially high-quality molecular description texts, are relatively scarce compared to general image-text data. This scarcity makes it inadequate for training a text encoder from scratch. Therefore, we initialize the BERT model with KV-PLM (Zeng *et al.* 2022). KV-PLM is a self-supervised learning model based on the BERT model. It is more suitable for processing text that describes molecular characteristics, as it is trained on a combination of molecular SMILES strings and molecular description texts.

The text encoder generates feature representations for text description by tokenizing words and projecting them to word embeddings $W_{0}=\left[w_{0}^{1},w_{0}^{2},\ldots ,w_{0}^{n_{t}}\right]\in R^{n_{t}\times d_{t}}$. At each stage, $W_{i}$ is input to (i+1)-th Transformer layer $L_{i+1}$ of text description encoder:

(9) $$W_{i}=L_{i}(W_{i-1}),\;i=1,2,\ldots ,K_{t}.$$

where $K_{t}$ represents the total number of layers in the text encoder, $K_{t}=12$.

### 2.5 Multi-modal contrastive pretraining

After obtaining two representations of drug molecules using a text encoder and a graph encoder, we apply a contrastive learning framework to align the representations of the same drug molecule, enhancing the understanding of its characteristics. Let $\left\{\left(g_{1},t_{1}\right),\left(g_{2},t_{2}\right),\ldots ,\left(g_{N},t_{N}\right)\right\}$ represent a batch of molecular graph-text pairs, where the molecular graph structure $g_{i}$ and the corresponding molecular description text $t_{i}$ constitute a positive pair $\left(g_{i},t_{i}\right)$, while it forms negative pairs ${\left(g_{i},t_{j}\right)}_{i\neq j}$ with different molecular description texts $t_{j}$.

Given the molecular graph structure representation $h^{K}$ and the molecular description text representation $w_{K}$, we use a Multilayer Perceptron (MLP) as the projection network to project both types of representations into the same dimensional feature space, as shown in the following formula.

(10) $$z^{G}=\mathrm{GraphProj}(h^{K}),\;z\in R^{d_{g}}.$$

 

(11) $$z^{T}=\mathrm{TextProj}(w_{K}),\;z\in R^{d_{t}}.$$

We denote the projected feature representation of the *i*th molecular graph as $z_{i}^{G}$, and the corresponding feature representation of the molecular description text as $z_{i}^{T}$. We adopt structure-text contrastive loss, which aims to bring samples from different modalities with the same semantic information closer in the feature space, while pushing apart samples with different semantic information (Fang *et al.* 2024). The loss function is defined as

(12) $$l_{g2t}^{i}=-\log\frac{\;\text{exp}\;\left(\mathrm{sim}\left(z_{i}^{G},z_{i}^{T}\right)/\tau \right)}{\sum_{j=1}^{N}exp\left(\mathrm{sim}\left(z_{i}^{G},z_{j}^{T}\right)/\tau \right)}$$

 

(13) $$l_{t2g}^{i}=-\log\frac{\;\text{exp}\;\left(\mathrm{sim}\left(z_{i}^{T},z_{i}^{G}\right)/\tau \right)}{\sum_{j=1}^{N}exp\left(\mathrm{sim}\left(z_{i}^{T},z_{j}^{G}\right)/\tau \right)}$$

 

(14) $$L=\frac{1}{2}\left(\sum_{i=1}^{N}l_{g2t}^{i}+\sum_{i=1}^{N}l_{t2g}^{i}\right)$$

where sim(·,·) denotes cosine similarity, and τ is the temperature hyperparameter, which is set to 0.1.

## 3 Results

### 3.1 Pretraining details

We use the PC200K dataset for pretraining, which contains approximately 200 000 molecular graph–text pairs collected from PubChem. We use Graphormer with 12 layers and a 768-dimensional hidden size as the molecular graph structure encoder. We select BERT as the text encoder. Molecular graph representations and text representations are projected into the same feature space using two multi-layer perceptrons, each of whose output dimension is 256. A summary of input and output dimensions for each module is provided in Table 2, available as supplementary data at *Bioinformatics* online.

**Table 2.** Performance comparison in molecular toxicity prediction.

Model	Ames ↑	hERG ↑	DILI ↑	LD50 ↓
KV-PLM	67.6 ± 0.6	65.2 ± 0.8	77.5 ± 1.4	0.859 ± 0.015
MoMu	80.6 ± 0.4	80.8 ± 0.4	87.3 ± 0.9	0.773 ± 0.008
MoleculeSTM	82.4 ± 0.5	83.4 ± 0.4	85.5 ± 1.3	0.770 ± 0.012
MOLEBLEND	82.5 ± 0.3	85.1 ± 0.7	88.6 ± 1.2	0.745 ± 0.009
**MolPrompt**	**82.7** ± **0.7**	**87.8** ± **0.5**	**90.0** ± **1.1**	**0.711** ± **0.011**

Bold values indicate our model and correspond to the best performance.

Before pretraining, we initialize the Graphormer with graphormer-base-pcqmv1, which is the checkpoint of Graphormer pretrained on the PCQM4M-LSC dataset, and initialize BERT with the checkpoint of KV-PLM. In the pretraining stage, we employ the AdamW optimizer with a learning rate of 1e−4 and a weight decay of 1e−5 for 50 epochs to pretrain MolPrompt. The batch size is set to 16.

### 3.2 Baselines

We select a set of baselines for comprehensive comparison with MolPrompt, including the latest multimodal molecular structure-text contrastive learning pretraining methods, namely KV-PLM (Zeng *et al.* 2022), MoMu (Su *et al.* 2022), MoleculeSTM (Liu *et al.* 2023a), MolCA (Liu *et al.* 2023b), and MolFM (Luo *et al.* 2023). We also consider MOLEBLEND (Yu *et al.* 2023), a multimodal molecular structure pretraining model that integrates both 2D and 3D molecular structures but does not incorporate textual modality. Detailed descriptions of these baselines are provided in Section 4, available as supplementary data at *Bioinformatics* online.

### 3.3 Molecule-text cross-modal retrieval

To validate whether MolPrompt effectively captures the inherent relationships between molecular graph structures and molecular description texts, we applied it to the molecule-text cross-modal retrieval task, which comprises two subtasks: M2T and T2M. The detailed retrieval procedures for both subtasks are described in Section 5, available as supplementary data at *Bioinformatics* online.

We evaluated the molecular graph-text cross-modal retrieval performance of MolPrompt on the PCDes (Zeng *et al.* 2022), MoMu (Su *et al.* 2022) and PubChem-Test datasets. Detailed descriptions of these datasets are provided in Section 5, available as supplementary data at *Bioinformatics* online. To avoid the risk of data leakage, we ensure that none of the samples from the three datasets are included during the pretraining stage. We conduct experiments on zero-shot retrieval scenarios, which directly test the generalization ability of pre-trained models without finetuning.

We perform retrieval in randomly sampled batches with a batch size of 64 and assess the retrieval performance using Recall@20 (R@20) and Top-1 Accuracy (Acc). Specifically, R@20 measures the frequency with which the correct match appears within the top 20 most similar results, while Acc denotes the percentage of queries for which the top-1 retrieved result is the correct match. Comparative results of various methods are presented in Table 1 and Table 3, available as supplementary data at *Bioinformatics* online. On the PCDes dataset, our method MolPrompt achieves the highest Recall@20 score of 88.2 in the M2T task, outperforming the best baseline model, MolCA, by 3.0%. In the T2M task, our method also achieves the highest Recall@20 score of 86.5, representing a significant 5.1% improvement compared to MolCA. On the MoMu dataset, our method demonstrates exceptional performance, achieving at least a 3.3% improvement in both M2T and T2M Recall@20 compared to the most competitive benchmark. On the PubChem-Test dataset, MolPrompt exhibits competitive performance, with both Top-1 Accuracy and Recall@20 significantly surpassing those of baseline models. These results underscore the strong generalization capability of our method in cross-modal retrieval tasks. It exhibits significant advantages in both cross-dataset and cross-task generalization, further verifying its effectiveness and robustness in molecule-to-text and text-to-molecule retrieval tasks. Overall, our method not only achieves leading performance on individual tasks but also maintains consistent performance across different datasets, emphasizing its applicability and stability.

Furthermore, a comparison between MolPrompt and its variant, MolPrompt-w/o prompt, reveals the contribution of knowledge prompts. On the PCDes dataset, MolPrompt achieves relative improvements of 1.7% in M2T and 3.5% in T2M tasks. Similar consistent improvements are also observed on the MoMu and PubChem-Test datasets, demonstrating the robustness of knowledge prompts integration. These improvements underscore the pivotal role of embedding domain-specific physicochemical priors into the graph encoder via text-based molecular knowledge prompts. This approach enriches molecular semantics and improves cross-modal alignment between molecular graphs and their textual descriptions by facilitating early interaction between graph topologies and textual property information.

To more clearly demonstrate the superiority of our model in the molecular cross-modal retrieval task, we illustrate two retrieval examples using 4-Hydroxybenzenesulfonic acid and 1,3-Propane Sultone, drawn from the PCDes dataset, as query molecules to retrieve the corresponding molecular description texts. The retrieval results and detailed analysis are provided in Fig. 1 and Section 5, available as supplementary data at *Bioinformatics* online.

### 3.4 Molecular property prediction

Understanding molecular properties is fundamental in drug discovery and material science, as it provides insights into the behavior and potential applications of chemical compounds. To evaluate the quality of learned molecular representations and the transferability of the pre-trained molecular graph encoder of MolPrompt, we apply MolPrompt to molecular property prediction. We solely utilize the molecular graph encoder of MolPrompt to generate fixed-length feature embedding for the molecule. Then we attach a prediction head (a linear layer) on top of the molecular graph encoder, which is used to predict whether the molecule possesses a target property or to infer the molecule’s response to the target property based on the learned molecular representation.

We conduct experiments on eight widely used datasets from MoleculeNet (Wu *et al.* 2018), including BBBP, Tox21, ToxCast, SIDER, ClinTox, MUV, HIV, and BACE, involving molecular physiological effects and biophysical interactions. We employ the Scaffold split method, allocating the data into training, validation, and test sets in an 8:1:1 ratio.

**Figure 2.**
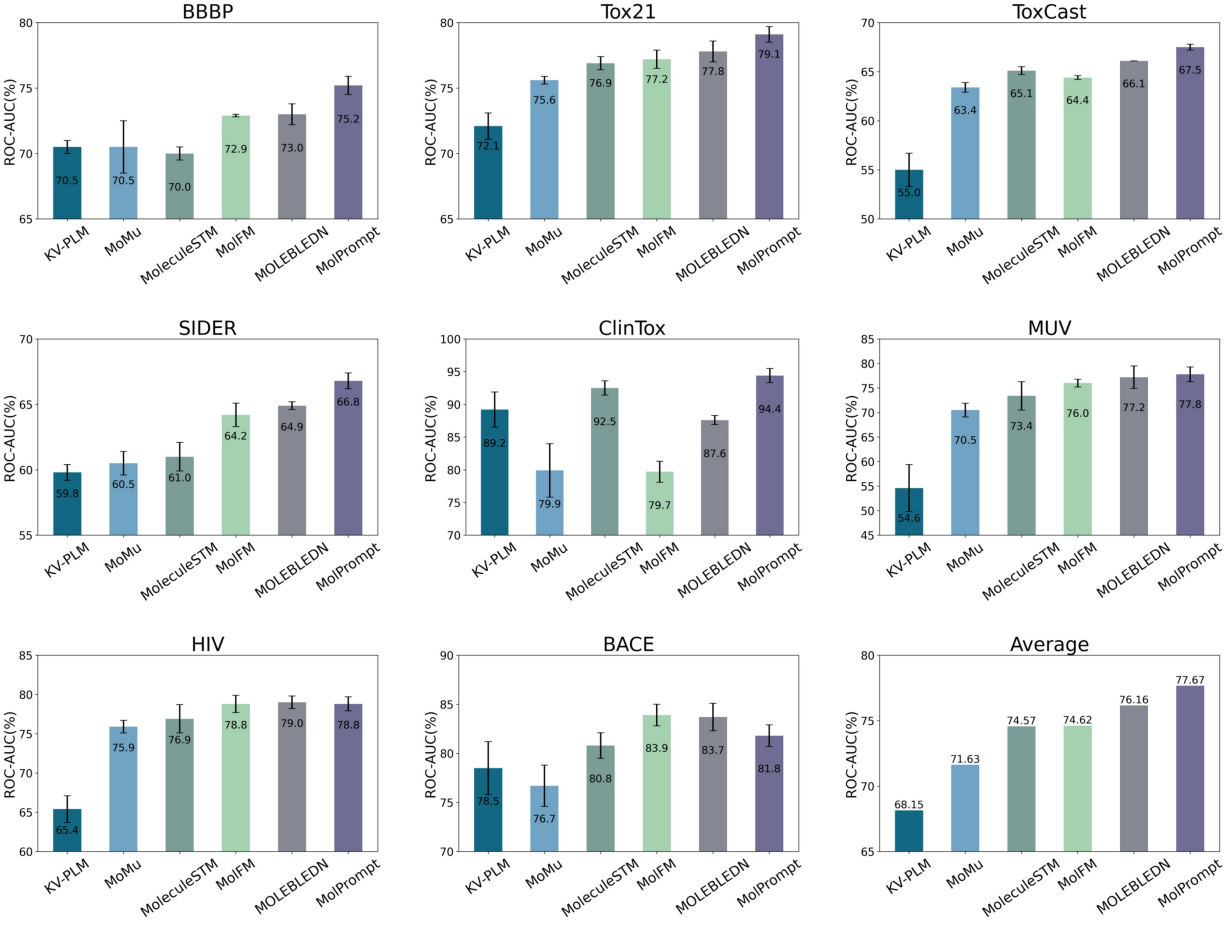
Performance comparison in molecular property prediction. We present the performance of MolPrompt compared to baseline models on eight datasets from MoleculeNet Wu *et al.* (2018), including BBBP, Tox21, ToxCast, SIDER, ClinTox, MUV, HIV, and BACE. ROC-AUC is used as the evaluation metric. MolPrompt consistently outperforms other methods on most datasets and achieves the best average performance.

We fine-tune the molecular graph structure encoder of MolPrompt, initialized with pre-trained model weights, on the train set of each dataset. We compare MolPrompt with baseline models that adopt different pre-training methods. We conduct experiments 10 times and report the mean and standard deviation of ROC-AUC scores (%), as shown in Fig. 2. The experimental results indicate that our model performs exceptionally well on classification tasks in physiology and biophysics, achieving the highest scores across 6 datasets, excluding HIV and Bace. On average, MolPrompt outperforms the best baseline, MOLEBLEND, by 2.0%, and surpasses the second-best model, MolFM, by 4.1%, demonstrating its effectiveness. MolPrompt significantly improves prediction performance in downstream tasks, with the prediction accuracy on the ClinTox dataset notably exceeding 94%. The experimental results comprehensively demonstrate that MolPrompt significantly outperforms the baseline models across various datasets, achieving remarkable performance in classification tasks related to physiology and biophysics, and delivering superior prediction accuracy in downstream tasks.

### 3.5 Molecular toxicity prediction

Toxicity prediction is crucial in the pharmaceutical industry as it helps to identify potential harmful effects of compounds early in the drug discovery process. To evaluate the performance of the molecular graph encoder of MolPrompt on the Molecular Toxicity Prediction task, we choose four datasets related to molecular toxicity from TDCommons (Huang *et al.* 2021)—Ames, hERG, DILI, and LD50. Detailed descriptions of these datasets are provided in Section 6, available as supplementary data at *Bioinformatics* online.

Similar to the molecular property prediction task, we only utilize the molecular graph encoder of MolPrompt to generate fixed-length feature embeddings for the molecules. Subsequently, we append a prediction head (comprising a linear layer) atop the molecular graph encoder. We fine-tune the molecular graph structure encoder of MolPrompt, initialized with pre-trained model weights, on the train set of each dataset. We undertake experiments ten times and present the mean and standard deviation of ROC-AUC scores (%) for three classification datasets (where higher scores indicate better performance) and RMSE for LD50 (where lower scores indicate better performance). The experimental outcomes are displayed in Table 2, with the optimal scores for each dataset emphasized in bold. Additionally, we evaluate the model’s robustness under low-resource settings, with details provided in Section 6 and Table 6, available as supplementary data at *Bioinformatics* online.

We benchmark our model against KV-PLM, MoMu, MoleculeSTM and MOLEBLEND. The experimental results reveal that our model surpasses others in predicting molecular toxicity, attaining the peak scores across all test tasks and datasets.

Activity cliffs represent a pivotal concept in medicinal chemistry, highlighting the intriguing phenomenon where molecules with closely related structures can exhibit markedly different biological activities. This phenomenon underscores the intricate interplay between molecular structure and properties, making it especially pivotal in drug design. To comprehensively illustrate the predictive prowess of our model, we meticulously select a pair of molecules that are structurally similar yet possess opposing properties from the test set of hERG for a detailed case study, visualizing the activity cliffs as depicted in Fig. 2a, available as supplementary data at *Bioinformatics* online. Our observations reveal that MolPrompt adeptly identifies these crucial substructures that differentiate the activity cliffs, demonstrating its proficiency in recognizing the distinctive features of molecules with varying properties.

By visualizing the attention maps, we can infer which features are most relevant to the MolPrompt’s decision-making process. We present a comparative visualization featuring two attention maps, as shown in Fig. 2b, available as supplementary data at *Bioinformatics* online. Despite the minimal structural differences between the two molecules, the attention weight distributions for Mol A and Mol B show clear distinctions, especially in higher layers. Several regions with notable differences are highlighted with red boxes. By mapping atomic positions derived from each molecule’s SMILES sequence onto the corresponding attention maps, we identify the spatial correspondence between atomic features and attention patterns. Notably, the subtle structural differences between the two molecules are distinctly localized within the red-highlighted regions of the attention maps, demonstrating MolPrompt’s capacity to sensitively capture fine-grained molecular variations. Additionally, attention gradually shifts from a local feature focus in lower layers to more global attention in higher layers, further demonstrating MolPrompt’s ability to learn key, global molecular representations.

### 3.6 Impact and interpretability of knowledge prompts

To evaluate the efficacy of incorporating knowledge prompts constructed from molecular descriptors into the molecular graph structure encoder of our model, we undertake ablation experiments. In Fig. 3a, available as supplementary data at *Bioinformatics* online, “w/o prompt” signifies our model without the inclusion of the knowledge prompt. This variant is pre-trained from scratch and subsequently fine-tuned for the task of molecular toxicity prediction. Examining the results presented in Fig. 3a, available as supplementary data at *Bioinformatics* online, we can deduce the following conclusions:

The complete model, which seamlessly integrates both the spatial structure information of the molecule and the pertinent chemical and physical property information imparted by the knowledge prompt, demonstrates superior performance across four datasets in the context of molecular toxicity prediction. Notably, when the knowledge prompt is omitted from our model, there is a notable decrease in ROC-AUC scores on the Ames, hERG, and DILI datasets. Similarly, there is a pronounced decline in regression performance on the LD50 dataset. These findings underscore the pivotal role of the knowledge prompt in enhancing the predictive capabilities of our model.

Furthermore, we are interested in the potential of knowledge prompts to provide domain-specific interpretability. We provide visualization examples of molecular descriptors of a pair of molecules from the hERG public database, as shown in Fig. 3b, available as supplementary data at *Bioinformatics* online. Consistent with literature (Yu *et al.* 2016, Lee and Yoo 2025), hERG inhibition (i.e. blocker) is positively correlative to molecular weight (MolWT), and negatively correlated to topological polar surface area (TPSA). This presents a new perspective, illustrating how knowledge prompts constructed from molecular descriptors bridge the gap between contrastive learning pre-training and downstream tasks.

To vividly demonstrate the representative prowess of MolPrompt, we utilize T-SNE (Van der Maaten and Hinton 2008) to visualize the molecular representations learned by MoMu, MoleculeSTM, MolPrompt, and MolPrompt-w/o prompt on the hERG dataset. The visualization procedures are detailed in Section 8, available as supplementary data at *Bioinformatics* online. As evident in Fig. 3c, available as supplementary data at *Bioinformatics* online, all four models achieve a considerable separation of molecular properties. However, MolPrompt stands out by producing the most distinct and clear classification boundaries. In Fig. 3d, available as supplementary data at *Bioinformatics* online, compared to the other three models, the molecular representations learned by MolPrompt exhibit a more uniform distribution, with the density estimation curve being noticeably less sharp. This underscores the capability of MolPrompt to mitigate the scarcity of detailed semantic information pertaining to molecular attributes by incorporating knowledge prompts derived from molecular descriptors. Consequently, MolPrompt captures molecular representations with greater precision by harmoniously integrating both the chemical and physical properties of the molecules. This further substantiates the effectiveness of MolPrompt in enhancing the representation of molecular data.

### 3.7 Uncovering potentially effective FGFR1 inhibitors

Fibroblast growth factor receptor 1 (FGFR1), implicated in various cancer types, has been extensively investigated as a target for antitumor therapy (Acevedo *et al.* 2007). We conducted evaluation tests, drug repositioning, and docking analyses targeting FGFR1 to validate the effectiveness of MolPrompt in real-world drug discovery scenarios.

To facilitate the identification of potent inhibitors against FGFR1, we collected 12,461 molecules with experimentally determined potency against FGFR1 from previous patents and research, measured in terms of the negative logarithm of the half maximal inhibitory concentration (pIC50). We comprehensively evaluated the prediction performance of MolPrompt on this dataset. Detailed comparison results are provided in Fig. 4a, available as supplementary data at *Bioinformatics* online. These results validated the superior generalizability and reliability of MolPrompt in the prediction of FGFR1 inhibitors.

We used MolPrompt to identify potential FGFR1 inhibitors through drug repositioning. Specifically, we first obtained 4,214 drugs that are FDA-approved or have passed clinical phase 1 (denoted as the FDA&PP1 dataset) from the website of Selleck Chemicals (https://www.selleckchem.com). We used MolPrompt fine-tuned on the FGFR1 inhibitors pIC50 dataset, to make predictions for the molecules from the FDA&PP1 dataset. Next, we visualized the molecular representations of molecules from the FGFR1 inhibitors pIC50 dataset and FDA&PP1 dataset derived from MolPrompt, as shown in Fig. 4b, available as supplementary data at *Bioinformatics* online. Furthermore, we selected the top ten predicted inhibitors identified during drug repurposing, as shown in Fig. 4c, available as supplementary data at *Bioinformatics* online. Among them, eight molecules have been experimentally validated as high-affinity or effective FGFR1 inhibitors. For example, erdafitinib, is an orally active small molecule with potent tyrosine kinase inhibitory activity against all four FGFR family members and selectivity versus other highly related kinases (Perera *et al.* 2017).

To strengthen our findings, we further conducted docking analyses for the top ten predictions from MolPrompt. Autodock Vina (Eberhardt *et al.* 2021), a widely used docking software, was employed for these tests. The reference protein-ligand structure (PDB ID: 5A4C (Klein *et al.* 2015)) guided the identification of the binding pocket. As depicted in Fig. 4d, available as supplementary data at *Bioinformatics* online, all the molecules achieved docking energies below -7 kcal/mol, a commonly used threshold for drug-like molecules (Chang *et al.* 2007), signifying the substantial potential for these molecules as FGFR1 inhibitors. Additionally, we conducted an in-depth analysis of the protein-ligand interactions for all the molecules that had not been reported in the literature using a widely applied protein-ligand interaction profiler named PLIP (Adasme *et al.* 2021). Figure 4e, available as supplementary data at *Bioinformatics* online, illustrates the protein-ligand interaction profile of the ligand PLX-4720 with the protein FGFR1. Further interaction analysis details are provided in Section 9, available as supplementary data at *Bioinformatics* online. These observations showcased that the molecules can tightly bind to FGFR1, validating the reliability of the docking results. Collectively, these results highlighted the superior ability of MolPrompt to identify potential inhibitors for FGFR1.

## 4 Conclusion

In this study, we present MolPrompt, a multimodal molecular pre-training framework that systematically integrates domain-specific knowledge through natural language prompts derived from physicochemical descriptors. By embedding these priors into the molecular graph encoder, MolPrompt enables early-stage interaction between topological structures and semantic properties, facilitating the learning of structurally grounded and semantically enriched molecular representations. This unified design improves the model’s ability to capture subtle intermodal dependencies between graph-based and sequence-based modalities, which is essential for accurate and generalizable molecular modeling.

MolPrompt demonstrates strong generalization and transferability across diverse downstream tasks. In molecule–text cross-modal retrieval, it achieves state-of-the-art performance, highlighting its capacity to semantically align structural and textual modalities. For molecular property prediction, MolPrompt consistently outperforms all baseline and advanced models, achieving the highest scores on six out of eight datasets, reflecting its superior capability in physiological and biophysical classification tasks. In molecular toxicity prediction, it maintains high accuracy, with case studies revealing precise attention to activity cliffs, which are regions associated with abrupt shifts in potency. Furthermore, MolPrompt demonstrates practical utility in drug discovery, exhibiting its capability to uncover potential inhibitors targeting the anticancer protein FGFR1.

Looking forward, future research will aim to develop more advanced architectures and explore the incorporation of additional modalities, with the goal of constructing a unified and comprehensive multimodal foundation model to further advance molecular understanding and prediction.

## Supplementary Material

btaf466_Supplementary_Data

## Data Availability

The data underlying this article are available in the article and in its online supplementary material.
