# Supplementary material for: MolPrompt: improving multi-modal molecular pre-training with knowledge prompts
Source: Bioinformatics. 2025 Aug 23;41(9):btaf466. doi: 10.1093/bioinformatics/btaf466 (PMC12448219; doi:10.1093/bioinformatics/btaf466)
Supplement: btaf466_Supplementary_Data [file btaf466_supplementary_data.pdf]

1 Data Collection

Advancements in computational biology have catalyzed the development of a suite of publicly accessible drug discovery databases, exemplified by PubChem (Kim et al., 2021), ChEMBL (Mendez et al., 2019), DrugBank (Wishart et al., 2018), and several others. PubChem stands out as one of the most extensive public molecular databases, serving as an open chemical information repository. It meticulously archives molecular structures, properties, bioactivities, and ancillary reference data for a comprehensive collection exceeding 100 million compounds. At present, PubChem has emerged as an indispensable resource for computational drug discovery and design endeavors.

We used the PubChem database as our data source, employing the PUG-View (Kim et al., 2019) web service to programmatically access and download data. We extracted the molecular CID, SMILES strings, and molecular descriptions, compiling a dataset of approximately 200K molecules. Using the smiles2graph function from OGB (Hu et al., 2020), we converted the SMILES strings into molecular graph structures. Most molecular descriptions begin with common names or International Union of Pure and Applied Chemistry (IUPAC) names. Following the methods of existing studies (Liu et al., 2023b; Luo et al., 2023; Liu et al., 2023a), we standardized these descriptions to a uniform text template ("This molecule is..."). To prevent any potential data leakage, molecules whose SMILES strings were identical to those in the downstream evaluation datasets were first removed from the pre-training dataset. To further eliminate structural redundancy beyond SMILES duplication, InChIKey-based deduplication was conducted between the pre-training and evaluation sets, confirming that no molecular identities overlapped across these datasets. We then matched each molecule's graph structure with its corresponding description based on the CID, resulting in a dataset called PC200K, which consists of approximately 200K molecular graph-structure-text pairs.

2 Details of Molecular Descriptors in Knowledge Prompts

Table S1. Explanation of molecular descriptors in knowledge prompts.

Molecular Descriptor	Description
MolWt	The sum of the relative atomic masses of all atoms in a molecule, typically measured in Daltons (Da).
MolLogP	The logarithm of a molecule's partition coefficient between octanol and water, which can serve as an indicator of its lipophilicity or lipid-solubility, and hydrophilicity or water-solubility.
NumRotatableBonds	The number of single bonds in a molecule that are capable of freely rotating, which is closely associated with the molecule's flexibility and its inherent degrees of freedom.
TPSA	The sum of the surface areas of all polar atoms within a molecule, frequently employed in the prediction of ADME (Absorption, Distribution, Metabolism, and Excretion) properties of pharmaceutical compounds.
NumHDonors	The total number of hydrogen bond donors in a molecule, typically associated with atoms such as nitrogen or oxygen that can donate hydrogen atoms to form hydrogen bonds, and is important in predicting molecular interactions and solubility.
NumHAcceptors	The total number of hydrogen bond acceptors in a molecule, typically associated with electronegative atoms like nitrogen or oxygen that can accept hydrogen atoms to form hydrogen bonds, and is important in predicting molecular interactions and solubility.
RingCount	The total number of rings in a molecule, representing the number of cyclic structures, and is an important descriptor for evaluating molecular stability, rigidity, and overall structural complexity.
NumAromaticRings	The total number of aromatic rings in a molecule, which is important in evaluating a molecule's stability, electronic properties, and aromaticity.
FractionCSP3	The proportion of carbon atoms in a molecule that are sp^3 hybridized, reflecting the degree of saturation and molecular flexibility, and is important for predicting the molecule's reactivity and steric properties.
BalabanJ	A topological index that quantifies the molecular complexity based on the connectivity of atoms in a molecule, and is commonly used to assess molecular diversity, stability, and other structural properties.

We employ the RDkit (Bento et al., 2020) toolkit to extract molecular descriptors from molecular SMILES sequences. RDKit offers over 200 molecular descriptors, from which we select ten representative descriptors chosen based on their broad applicability and relevance to diverse molecular properties as the external chemical and physical property prior knowledge of the molecules. These include MolWt (molecular weight), MolLogP (molecular logP), NumRotatableBonds (number of rotatable bonds), TPSA (topological polar surface area), NumHDonors (number of hydrogen bond donors), NumHAcceptors (number of hydrogen bond acceptors), RingCount (ring count), NumAromaticRings (number of aromatic rings), FractionCSP3 (fraction of carbon atoms with three bonds to hydrogen - CSP3), and BalabanJ (Balaban's J index). These descriptors are not task-specific but are selected for their ability to represent fundamental physicochemical properties that broadly

impact molecular behavior and activity across a wide range of biological contexts. Our framework can be readily extended to incorporate additional descriptors as needed, enabling scalability for future work. A detailed explanation of each molecular descriptor is provided in Table S1.

3 Overview of Module Dimensions

We summarize the input and output dimensions of key modules in MolPrompt. Table S2 presents the dimensionality transformations across the knowledge prompt construction, molecular graph encoder, text encoder, and the corresponding projection networks, offering a clear overview of the data flow within the contrastive learning framework.

Table S2. Input and output dimensions of key modules in MolPrompt

Module	Input Dimension	Output Dimension
Knowledge Prompt Construction	$n_p \times 768$	768
Molecular Graph Encoder	$n \times 768$	768
Text Encoder	$n_t \times 768$	768
Molecular Graph Projector	768	256
Text Projector	768	256

4 Baselines

We select a set of baselines for comprehensive comparison with MolPrompt, including the latest multimodal molecular structure-text contrastive learning pretraining methods, namely KV-PLM (Zeng et al., 2022b), MoMu (Su et al., 2022), MoleculeSTM (Liu et al., 2023b), MolCA (Liu et al., 2023c), and MolFM (Luo et al., 2023). We also consider MOLEBLEND (Yu et al., 2023), a multimodal molecular structure pretraining model that integrates both 2D and 3D molecular structures but does not incorporate textual modality. We provide detailed descriptions of the baseline models used for comparison with MolPrompt. Beyond architectural differences, these models also differ in scale, with KV-PLM, MoleculeSTM, MolFM, and our proposed MolPrompt containing approximately 110 million, 112 million, 138 million, and 220 million parameters, respectively.

- KV-PLM (Zeng et al., 2022b) employs a self-supervised language model to learn meta-knowledge from large-scale molecular science data while exploring a novel application of the MLM task. By inserting SMILES strings into their corresponding molecular description texts, KV-PLM integrates two types of information within a unified language modeling framework to leverage text description for a deeper understanding of molecules.
- MoMu (Su et al., 2022) is a molecular multimodal foundation model that directly associates molecular graph structures with natural language descriptions, pre-trained on a large number of molecular graph-text pairs via contrastive learning strategy. MoMu employs two graph augmentation methods, namely node dropping and subgraph, to obtain two augmented graphs for each molecule.
- MoleculeSTM (Liu et al., 2023b) constructs a large-scale molecular graph-text dataset, adopting a contrastive learning strategy to jointly learn the molecular graph structure and text description of molecules.
- MolCA (Liu et al., 2023c) combines 2D molecular graph with language model, enableing the language model to understand molecular information based on both text and graph via the cross-modal projector. Specifically, the cross-modal projector is implemented as a Q-Former, which connects the representation space of the molecular graph encoder with the text space of the language model.
- MolFM (Luo et al., 2023) jointly learns molecular representations from three different modalities: molecular structure, biomedical text, and knowledge graph. MolFM not only captures the local information of molecules but also obtains global molecular knowledge through integrating the knowledge graph, thereby mining deeper associations in the complex network of molecular science.
- MOLEBLEND (Yu et al., 2023) blends atom relations represented by 2D and 3D structure modalities into one unified relation matrix for joint encoding, then recovers modality-specific information for 2D and 3D structures individually. By treating atom relationships as anchors, MOLEBLEND organically aligns and integrates visually dissimilar 2D and 3D modalities of the same molecule at fine-grained atomic level.

5 Molecule-Text Cross-Modal Retrieval

5.1 Molecule-Text Cross-Modal Retrieval Task Description

Molecule-text cross-modal retrieval task consists of two subtasks: molecule-to-text (M2T) and text-to-molecule (T2M). In the M2T task, the molecular graph encoder and text encoder of MolPrompt generate embeddings for all molecules and texts, respectively. Using the molecular graph structure embedding as a query, we compare it against the set of text embeddings and select the ones with the highest cosine similarity to identify the best match. Conversely, the T2M task follows the reverse process.

5.2 Molecule-Text Cross-Modal Retrieval Datasets

We evaluated the molecular graph-text cross-modal retrieval performance of MolPrompt on the PCDes (Zeng et al., 2022b), MoMu (Su et al., 2022) and PubChem-Test datasets. The PCDes dataset includes SMILES strings and corresponding molecular description texts for 15,000 molecules, while the MoMu dataset contains 5,562 molecular graph-text pairs. The PubChem-Test dataset consists of 3,000 graph–text pairs that we collected from PubChem, and which are excluded from the pre-training dataset.

Table S3. Performance comparison on the PubChem-Test dataset for cross-modal molecule-text retrieval tasks.

	**M2T**		**T2M**	
Model	M2T Acc	M2T R@20	T2M Acc	T2M R@20
KV-PLM	39.4	80.4	40.2	81.7
MoMu-S	44.9	81.6	42.6	80.5
MoMu-K	45.6	83.1	45.1	82.2
MoleculeSTM	49.3	84.9	51.3	85.8
MolCA	62.6	88.7	61.2	87.1
**MolPrompt-w/o prompt**	**63.8**	**89.1**	**62.5**	**87.8**
**MolPrompt**	**65.4**	**90.9**	**64.1**	**89.7**

Table S4. Recall@5 and Recall@10 on PCDes for M2T and T2M retrieval tasks.

Model	M2T R@5	M2T R@10	T2M R@5	T2M R@10
KV-PLM	37.9	45.7	37.3	45.3
MoMu-S	45.0	53.3	44.2	53.7
MoMu-K	45.4	53.8	44.9	54.2
MoleculeSTM	49.9	59.3	49.4	58.2
MolCA	51.8	60.1	52.6	60.9
**MolPrompt**	**52.6**	**63.3**	**53.2**	**63.5**

Table S5. Mean ± standard deviation of retrieval performance (Recall@20) over five random seeds. Statistical significance is assessed via paired t-tests against MolCA.

**Model**	**PCDes M2T**	**PCDes T2M**	**MoMu M2T**	**MoMu T2M**
MolCA	85.8 ± 0.5	82.1 ± 0.4	76.9 ± 0.4	73.0 ± 0.3
**MolPrompt**	$\mathbf{88.3 \pm 0.4}$	$\mathbf{86.3 \pm 0.3}$	$\mathbf{79.6 \pm 0.3}$	$\mathbf{76.7 \pm 0.4}$
**p-value**	< 0.01	< 0.01	< 0.01	< 0.01

5.3 Case Study

We illustrate two retrieval examples using 4-Hydroxybenzenesulfonic acid and 1,3-Propane Sultone, drawn from the PCDes dataset, as query molecules to retrieve the corresponding molecular description texts, as shown in Figure S1. We present the top three retrieval results of MoMu, MoleculeSTM and MolPrompt. In both retrieval examples, the top two retrieval results of both MoMu-K and MoleculeSTM fail to match the molecule, whereas the first retrieval result of MolPrompt accurately retrieves the description corresponding to the molecular graph.

6 Molecular Toxicity Prediction

6.1 Description of Toxicity Datasets

We choose four datasets related to molecular toxicity from TDCommons (Huang et al., 2021), a platform with AI-ready datasets and tasks for therapeutics, spanning the discovery and development of medicines, aiming to promote open science practices in drug research and support the application of machine learning and artificial intelligence in the field of drugs.

- Ames. Mutagenicity refers to the ability of a drug to induce genetic alterations. Drugs that can cause damage to DNA can lead to cell death or other severe side effects. The Ames experiment is currently the most widely used method for detecting molecular mutagenicity.
- hERG. Human ether-a-go-go related gene (hERG) is essential for the coordination of heartbeats. Drugs that block the hERG can cause severe adverse effects.
- DILI. Drug-induced liver injury (DILI) is a fatal liver disease caused by drugs and it has been the most common cause of drug marketing withdrawals due to safety concerns in the last decades (e.g. iproniazid, ticrynafen, benoxaprofen).
- LD50. Acute toxicity LD50 measures the most conservative dose that can cause lethal adverse effects. The higher the dose, the higher the lethality of the drug.

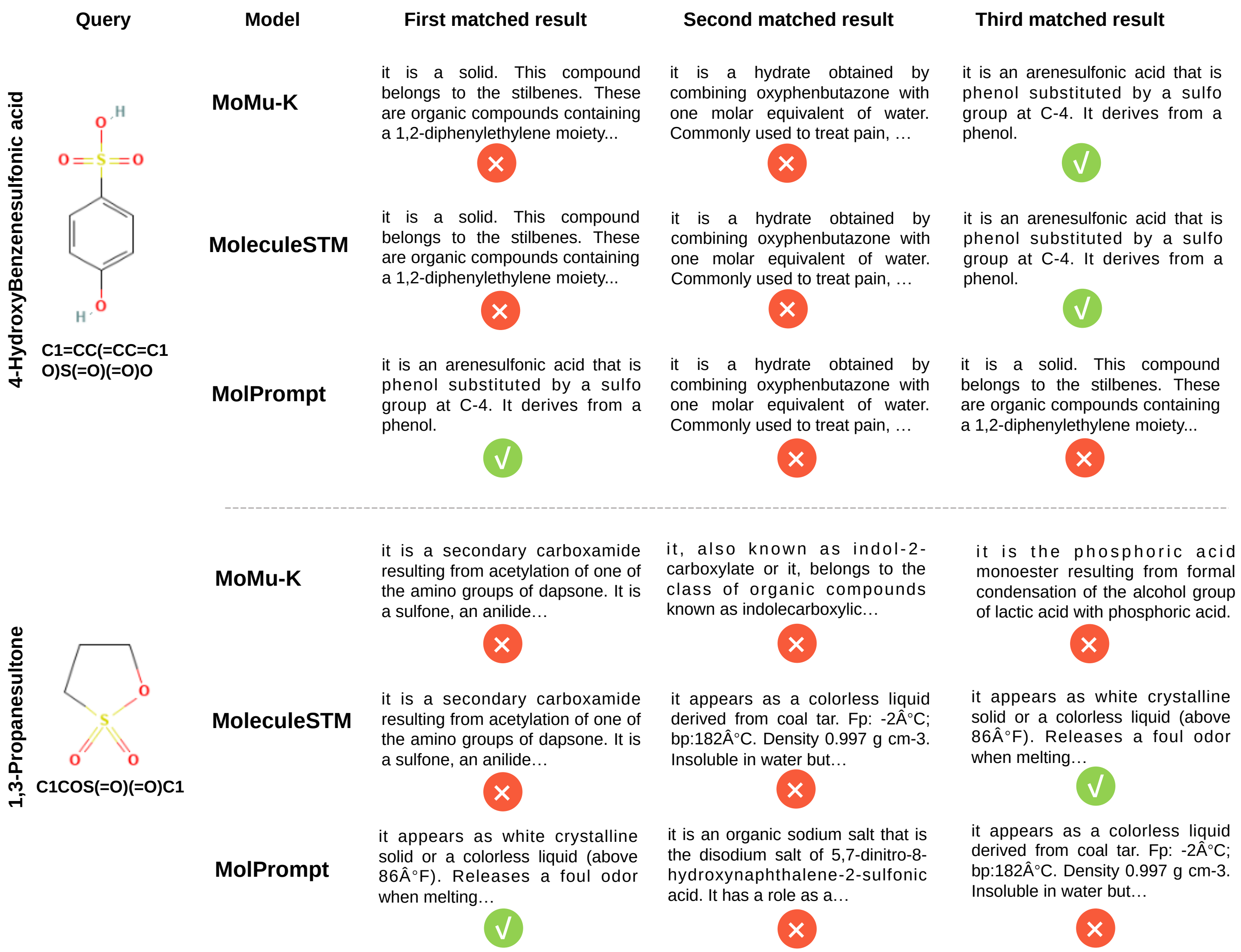

Figure S1. Case study of the molecule-to-text retrieval task. We illustrate two retrieval examples using 4-Hydroxybenzenesulfonic acid (top) and 1,3-Propane Sultone (bottom), drawn from the PCDes dataset, as query molecules to retrieve the corresponding molecular description texts.

6.2 Performance Comparison under Low-Resource Settings

To evaluate the robustness and generalization capability of different models under limited data conditions, we conduct experiments under low-resource settings. Specifically, based on the original 8:1:1 train/validation/test split, we randomly sample 20% of the training set to form a new, smaller training set, while keeping the validation and test sets unchanged. The performance results are summarized in Table S4. MolPrompt consistently outperforms baseline models across all four molecular toxicity prediction tasks, even under low-resource settings, demonstrating strong robustness and transferability.

Table S6. Performance comparison under low-resource settings in molecular toxicity prediction.

Model	Ames ↑	hERG ↑	DILI ↑	LD50 ↓
KV-PLM	59.7 ± 0.8	55.2 ± 1.3	75.8 ± 1.1	0.904 ± 0.010
MoMu	73.4 ± 0.6	72.0 ± 0.7	84.5 ± 0.6	0.827 ± 0.009
MoleculeSTM	74.1 ± 0.5	73.8 ± 0.9	84.3 ± 1.3	0.802 ± 0.012
MOLEBLEND	76.2 ± 0.9	77.1 ± 0.6	85.2 ± 0.6	0.795 ± 0.008
**MolPrompt**	**77.4 ± 0.6**	**79.4 ± 0.7**	**86.7 ± 0.7**	**0.772 ± 0.014**

7 Case Study and Attention Visualization on Activity Cliffs

To comprehensively illustrate the predictive prowess of our model, we meticulously select a pair of molecules (denoted as Mol A and Mol B) that are structurally similar yet possess opposing properties from the test set of hERG for a detailed case study, visualizing the activity cliffs as depicted in Figure S2a. The dashed circles in the figure denote the distinct substructures that lie within these activity cliffs. We present a comparative visualization featuring two attention maps: one from Mol A and the other from Mol B, as shown in Figure S2b. Each row from left to right represents the attention score matrix from lower to higher layers. For the attention maps, brighter yellow indicates higher attention, while darker colors represent lower attention.

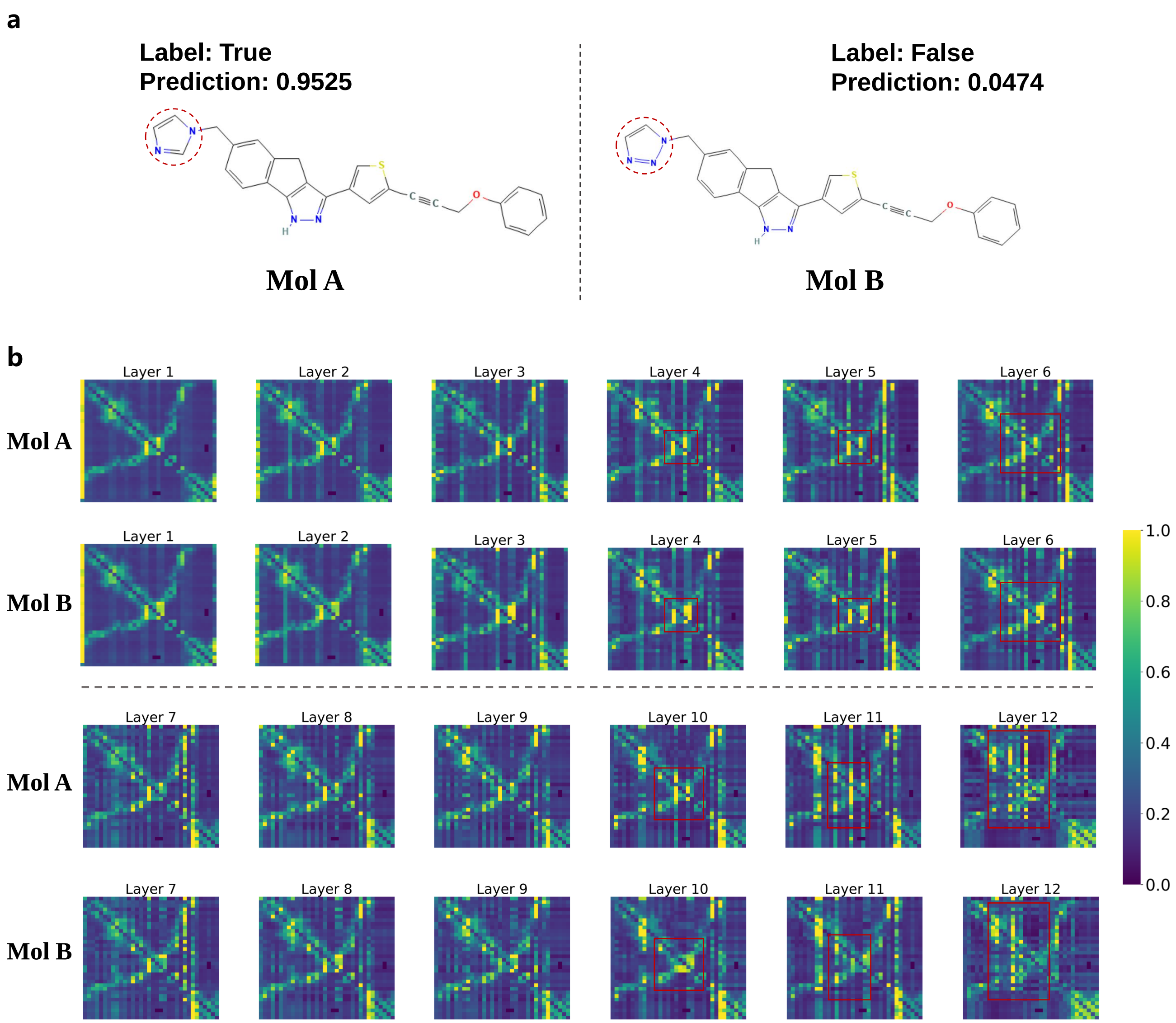

Figure S2. a. Case Study: the visualization of activity cliffs. Mol A blocks the hERG, which could lead to severe adverse effects, whereas Mol B does not block the hERG. The dashed circles in the figure denote the distinct substructures that lie within these activity cliffs. **b.** Visualization of attention maps from Mol A and Mol B. Brighter yellow indicates higher attention, while darker colors represent lower attention.

8 Impact and Interpretability of Knowledge Prompt

8.1 T-SNE Visualization

We utilize T-SNE (Van der Maaten and Hinton, 2008) to visualize the molecular representations learned by MoMu, MoleculeSTM, MolPrompt, and MolPrompt-w/o prompt on the hERG dataset. Here, purple dots signify positive labels, while green dots denote negative labels. Subsequently, we examine the uniformity of the molecular representations learned by these four models. We map the learned molecular representations onto the unit hypersphere $\mathcal{S}^1$ using T-SNE, and then visualize the density distributions of the representations on $\mathcal{S}^1$ using

non-parametric Gaussian kernel density estimation (KDE) in $\mathcal{R}^2$. We also show the density estimations of angles for each point on $\mathcal{S}^1$ to present the results more clearly.

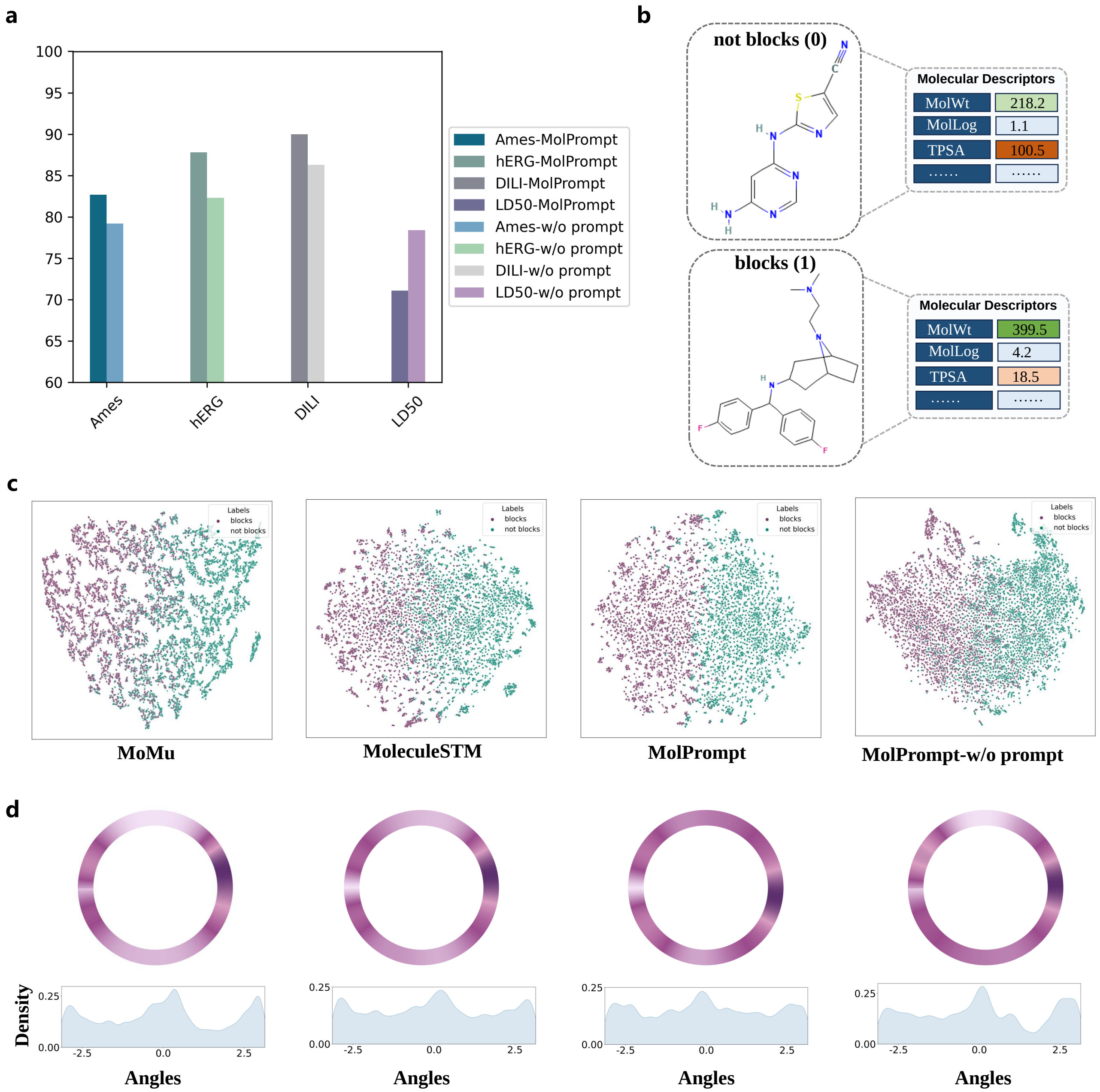

Figure S3. **a.** Ablation experiments: the pivotal role of the knowledge prompt in MolPrompt. **b.** Investigation of interpretability of knowledge prompts in MolPrompt. **c.** T-SNE visualization of the molecular representation space of MoMu, MoleculeSTM, MolPrompt, and MolPrompt-w/o prompt on the hERG dataset. **d.** Uniformity analysis: Molecular representation distributions are plotted with Gaussian KDE in $\mathcal{R}^2$ (darker colours indicate more points fall in the region), along with KDE on angles for a clearer presentation.

9 Uncovering Potentially Effective FGFR1 Inhibitors

9.1 Comparison on FGFR1 Inhibitor Prediction

We collected 12461 molecules with experimentally determined potency against FGFR1 from previous patents and research, measured in terms of the negative logarithm of the half maximal inhibitory concentration (pIC50). We comprehensively evaluated the prediction performance of MolPrompt on this dataset using scaffold splitting, with the data partitioned into training, validation, and test sets in an 8:1:1 ratio. To

prevent data leakage, we ensured that no molecules in this dataset overlapped with the pretraining dataset by removing SMILES duplicates and further performing InChIKey-based deduplication to eliminate structural redundancy. Comparison results are detailed in Figure S4a. The results demonstrated that MolPrompt significantly outperformed MoMu and MoleculeSTM, as well as five structure-based molecular pretraining approaches, including GEM (Fang et al., 2022), 3D Infomax (Stärk et al., 2022), MolFormer (Ross et al., 2022), ImageMol (Zeng et al., 2022a) and GraphMVP (Liu et al., 2021), in terms of Spearman's r and Pearson's r.

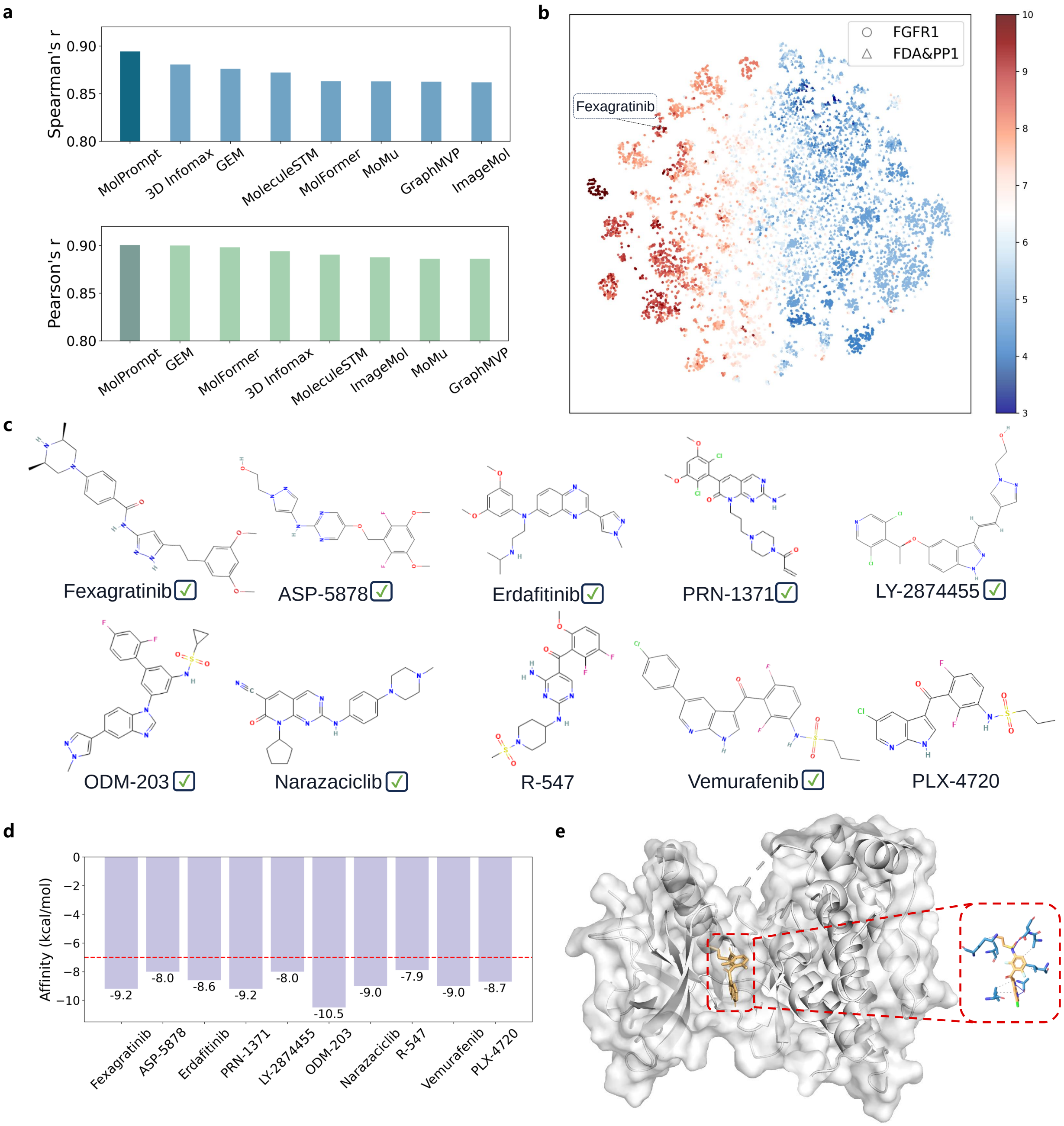

Figure S4. a. The performance of MolPrompt and baseline methods in predicting FGFR1 inhibitors is measured by Spearman's r and Pearson's r, respectively. **b.** Visualization of molecular representations of molecules from the FGFR1 inhibitors pIC50 dataset and FDA&PP1 dataset derived from MolPrompt. **c.** The top ten predictions of potential inhibitors against FGFR1 from the FDA&PP1 dataset derived from MolPrompt. The check symbols indicate that the molecules had been previously identified as inhibitors of FGFR1 in previous studies. **d.** The docking scores of the top ten molecules identified by MolPrompt as potential inhibitors against FGFR1, measured by Autodock Vina. A lower docking score (i.e., a more negative value) reflects a stronger predicted binding affinity between the ligand and the target protein. **e.** The interactions between PLX-4720 and FGFR1 profiled by PLIP. The protein ligand structure (PDB ID: 5A4C) was utilized as a reference for binding pocket identification.

9.2 Analysis of Protein–Ligand Interactions

We conducted an in-depth analysis of the protein-ligand interactions for all the molecules that had not been reported in the literature using a widely applied protein-ligand interaction profiler named PLIP (Adasme et al., 2021). Figure S4e illustrates the protein-ligand interaction profile of the ligand PLX-4720 with the protein FGFR1. The analysis revealed the formation of six hydrophobic interactions and four hydrogen bonds between the ligand and the protein. Among these interactions, the hydrogen bond formed with residues 641A and 564A were also reported in the reference protein-ligand structure (PDB ID: 5A4C (Klein et al., 2015)).

References

M. F. Adasme, K. L. Linnemann, S. N. Bolz, F. Kaiser, S. Salentin, V. J. Haupt, and M. Schroeder. Plip 2021: expanding the scope of the protein–ligand interaction profiler to dna and rna. *Nucleic acids research*, 49(W1):W530–W534, 2021.

A. P. Bento, A. Hersey, E. Félix, G. Landrum, A. Gaulton, F. Atkinson, L. J. Bellis, M. De Veij, and A. R. Leach. An open source chemical structure curation pipeline using rdkit. *Journal of Cheminformatics*, 12:1–16, 2020.

X. Fang, L. Liu, J. Lei, D. He, S. Zhang, J. Zhou, F. Wang, H. Wu, and H. Wang. Geometry-enhanced molecular representation learning for property prediction. *Nature Machine Intelligence*, 4(2):127–134, 2022.

W. Hu, M. Fey, M. Zitnik, Y. Dong, H. Ren, B. Liu, M. Catasta, and J. Leskovec. Open graph benchmark: Datasets for machine learning on graphs. *Advances in neural information processing systems*, 33:22118–22133, 2020.

K. Huang, T. Fu, W. Gao, Y. Zhao, Y. Roohani, J. Leskovec, C. W. Coley, C. Xiao, J. Sun, and M. Zitnik. Therapeutics data commons: Machine learning datasets and tasks for drug discovery and development. *arXiv preprint arXiv:2102.09548*, 2021.

S. Kim, P. A. Thiessen, T. Cheng, J. Zhang, A. Gindulyte, and E. E. Bolton. Pug-view: programmatic access to chemical annotations integrated in pubchem. *Journal of cheminformatics*, 11(1):56, 2019.

S. Kim, J. Chen, T. Cheng, A. Gindulyte, J. He, S. He, Q. Li, B. A. Shoemaker, P. A. Thiessen, B. Yu, et al. Pubchem in 2021: new data content and improved web interfaces. *Nucleic acids research*, 49(D1):D1388–D1395, 2021.

T. Klein, N. Vajpai, J. J. Phillips, G. Davies, G. A. Holdgate, C. Phillips, J. A. Tucker, R. A. Norman, A. D. Scott, D. R. Higazi, et al. Structural and dynamic insights into the energetics of activation loop rearrangement in fgfr1 kinase. *Nature communications*, 6(1):7877, 2015.

P. Liu, Y. Ren, and Z. Ren. Git-mol: A multi-modal large language model for molecular science with graph. *Image, and Text*, 2023a.

S. Liu, H. Wang, W. Liu, J. Lasenby, H. Guo, and J. Tang. Pre-training molecular graph representation with 3d geometry. *arXiv preprint arXiv:2110.07728*, 2021.

S. Liu, W. Nie, C. Wang, J. Lu, Z. Qiao, L. Liu, J. Tang, C. Xiao, and A. Anandkumar. Multi-modal molecule structure–text model for text-based retrieval and editing. *Nature Machine Intelligence*, 5(12):1447–1457, 2023b.

Z. Liu, S. Li, Y. Luo, H. Fei, Y. Cao, K. Kawaguchi, X. Wang, and T.-S. Chua. Molca: Molecular graph-language modeling with cross-modal projector and uni-modal adapter. *arXiv preprint arXiv:2310.12798*, 2023c.

Y. Luo, K. Yang, M. Hong, X. Y. Liu, and Z. Nie. Molfm: A multimodal molecular foundation model. *arXiv preprint arXiv:2307.09484*, 2023.

D. Mendez, A. Gaulton, A. P. Bento, J. Chambers, M. De Veij, E. Félix, M. P. Magariños, J. F. Mosquera, P. Mutowo, M. Nowotka, et al. Chembl: towards direct deposition of bioassay data. *Nucleic acids research*, 47(D1):D930–D940, 2019.

J. Ross, B. Belgodere, V. Chenthamarakshan, I. Padhi, Y. Mroueh, and P. Das. Large-scale chemical language representations capture molecular structure and properties. *Nature Machine Intelligence*, 4(12):1256–1264, 2022.

H. Stärk, D. Beaini, G. Corso, P. Tossou, C. Dallago, S. Günnemann, and P. Liò. 3d infomax improves gnns for molecular property prediction. In *International Conference on Machine Learning*, pages 20479–20502. PMLR, 2022.

B. Su, D. Du, Z. Yang, Y. Zhou, J. Li, A. Rao, H. Sun, Z. Lu, and J.-R. Wen. A molecular multimodal foundation model associating molecule graphs with natural language. *arXiv preprint arXiv:2209.05481*, 2022.

L. Van der Maaten and G. Hinton. Visualizing data using t-sne. *Journal of machine learning research*, 9(11), 2008.

D. S. Wishart, Y. D. Feunang, A. C. Guo, E. J. Lo, A. Marcu, J. R. Grant, T. Sajed, D. Johnson, C. Li, Z. Sayeeda, et al. Drugbank 5.0: a major update to the drugbank database for 2018. *Nucleic acids research*, 46(D1):D1074–D1082, 2018.

Q. Yu, Y. Zhang, Y. Ni, S. Feng, Y. Lan, H. Zhou, and J. Liu. Multimodal molecular pretraining via modality blending. *arXiv preprint arXiv:2307.06235*, 2023.

X. Zeng, H. Xiang, L. Yu, J. Wang, K. Li, R. Nussinov, and F. Cheng. Accurate prediction of molecular properties and drug targets using a self-supervised image representation learning framework. *Nature Machine Intelligence*, 4(11):1004–1016, 2022a.

Z. Zeng, Y. Yao, Z. Liu, and M. Sun. A deep-learning system bridging molecule structure and biomedical text with comprehension comparable to human professionals. *Nature communications*, 13(1):862, 2022b.
